# Biosynthetic Potential of *Streptomyces* Rationalizes Genome-Based Bioprospecting

**DOI:** 10.3390/antibiotics10070873

**Published:** 2021-07-19

**Authors:** Balasubramanian Cibichakravarthy, Polapass Arul Jose

**Affiliations:** 1 Koret School of Veterinary Medicine, The Robert H. Smith Faculty of Agriculture, Food and Environment, The Hebrew University of Jerusalem, Rehovot 761000, Israel; chakravarthy@mail.huji.ac.il; 2 Department of Entomology and Plant Pathology & Microbiology, The Hebrew University of Jerusalem, POB 12, Rehovot 761000, Israel

**Keywords:** *Streptomyces*, genome-mining, natural products, antibiotics

## Abstract

*Streptomyces* are the most prolific source of structurally diverse microbial natural products. Advancing genome-based analysis reveals the previously unseen potential of *Streptomyces* to produce numerous novel secondary metabolites, which allows us to take natural product discovery to the next phase. However, at present there is a huge disproportion between the rate of genome reports and discovery of new compounds. From this perspective of harnessing the enduring importance of *Streptomyces*, we discuss the recent genome-directed advancements inspired by hidden biosynthetic wealth that provide hope for future antibiotics.

## 1. Introduction

Actinobacteria are Gram-positive bacteria known for their robust biosynthetic potential to produce secondary metabolites [1,2,3]. In the 1940s, Waksman group at Rutgers University laid the prime foundation by discovering novel compounds such as actinomycin, streptothricin, and streptomycin [4]. After this first breakthrough, the initial thirty years are considered a “golden age” because most discoveries were achieved during that period, and following 30 years involved the sophistication of screening methods with few modifications to the commercial products [5]. During the 1990s, the one strain/many compounds (OSMAC) approach was initiated to reveal nature’s chemical diversity. This concept is based on the environment and cultivation conditions, single strains of bacteria often have the potential to produce a variety of differential natural products [6]. In the late 1990s, most pharmaceutical companies abandoned natural product discovery due to a lack of technologies to address rediscovery issues that impact investment returns [7]. On the other hand, the advent of combinatorial chemistry incited the discovery of a large array of structurally diverse compounds for biological activity screening. Consequently, large pharmaceutical companies realigned their strategies and proceeded with the mass withdrawal from new natural product discovery. Nevertheless, there is an absolute need for new antibiotics to treat infectious diseases. In recent years, genomics databases have become dumping grounds of genome data that widen the scientific landscape, and researchers are racing to keep up. While genome-wide studies heavily impact overall scientific contributions [8], their impact is anticipated in the field of microbial natural products research. The aim of this article is to summarize the importance of *Streptomyces* genomics and its hidden treasure troves for finding novel compounds based on genome-guided approaches.

## 2. Deluge of Genome Data

While global genome datasets upsurge with genomes of different organisms, specially evolved simple bacterial genomes are sequenced in relatively high numbers. This has led to the deposition of more than 0.2 million bacterial genomes in public archives. Among the diverse bacteria targeted for genome sequencing, members of the actinobacterial genus *Streptomyces* have gained special attention due to their versatility in bioactive secondary metabolite biosynthesis. Using classical cloning approaches, *Streptomyces coelicolor* was the first actinobacterium sequenced completely for its natural products, followed by *Streptomyces avermitilis*, an industrially potent bacteria [9,10]. Genome sequencing of these two streptomycetes revealed the presence of unsurpassed biosynthetic potential in their genomes and set up a future for genome-guided natural product searches. Successively, 1902 genome entries, including 217 complete genome assemblies, were found at the time of this article submission. While genomes of different bacteria are sequenced for taxonomic purposes, *Streptomyces* genomes are largely sequenced because of their targeted biosynthetic potential.

## 3. Genome Inspired Mining Tools

Large numbers of *Streptomyces* genomes have resulted in the development and use of bioinformatics tools, which are specifically geared towards the discovery of biosynthetic gene clusters (BGCs). Bacterial antiSMASH is a versatile genome-mining pipeline that allows users to rapidly identify, annotate, and analyze secondary metabolite biosynthesis gene clusters (Blin et al., 2019; e.g., Sinha et al., 2019; Kumar et al., 2020; Malik et al., 2020). Integrated and cross-linked with a large number of *in silico* secondary metabolite analysis tools, antiSMASH handled about 700,000 analyses, and remains the de facto standard for mining BGCs in genome sequences of *Streptomyces* [11]. As non-ribosomal peptide (NRP) and polyketide (PK) BGCs produce highly diverse natural products, several tools targeted genes of these clusters. For instance, NaPDos has been developed to analyze ketosynthase (KS) and condensation (C) domains from PKS and NRPS, respectively [12]. The domain organization of PKS and NRPS were used for analyzing polyketide synthases, PKSs/non-ribosomal peptide synthetases (NRPSs) analysis website [13]. The amino acid selectivity of NRPS is predicted via NRPSpredictor, which is one of the most useful computational tools [14]. RiPPMiner is the tool to analyze the ribosomally synthesized and post translationally modified peptides (RiPPs) of BGCs [15]. In addition, other tools that benefits genome mining are the Integrated Microbial Genomes Atlas of Biosynthetic Gene Clusters (IMG-ABC) created by the Joint Genome Institute (JGI). The Minimum Information about a Biosynthetic Gene cluster (MIBiG) database maintained by Genomic Standards Consortium is also a useful database available for identifying candidate BGCs [16]. However, limited novel compounds of biological interest have been discovered because under pure culture conditions of *Streptomyces*, most of the secondary metabolite biosynthetic gene clusters are apparently silent (cryptic). In addition, secondary metabolites are assembled by mega-enzyme complexes, the expression of which requires a significant amount of energy and resources. Integration of genome-mining tools along with classical biological screening (see the review [17]) may boost the discovery of novel compounds.

## 4. Genome Inspired Efforts Nourish Natural Products Research

**Figure 1:**
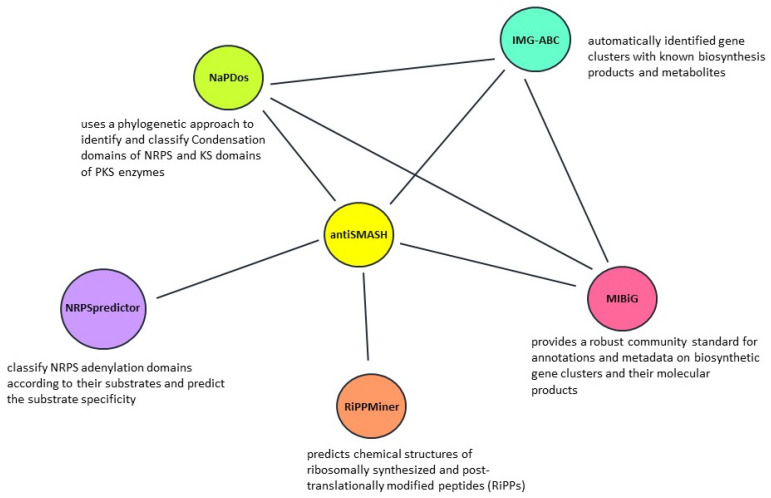
A conceptual framework of tools used for *Streptomyces* genome mining for the discovery of biosynthetic gene clusters (BGCs).

**Figure 2:**
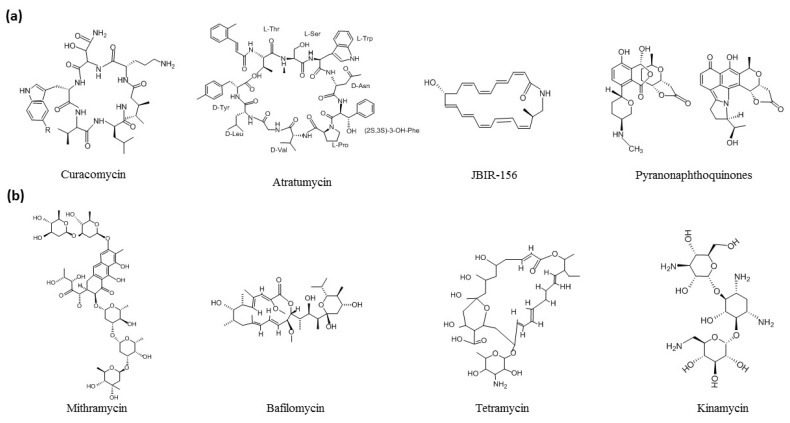
Illustration of chemical structures of notable compounds discovered through genome-directed approaches. (**a**) Novel compounds. (**b**) Over expressed compounds.

Recent promising strategies uniting bioinformatics-driven genome mining [18], controlled expression of novel biosynthetic gene clusters [19], and high-resolution metabolic profiling [20] are receiving substantial attention. Based on these, a conceptual network model emphasizing the relationship between genome mining tools used for the discovery of novel biosynthetic gene clusters are represented in Figure 1. Genome directed research favors the discovery of novel compounds and the overexpression of known bioactive compounds. For instance, in the large scale genome mining of about 10,000 actinomycetes, 11 previously undescribed phosphonic acids were discovered [21]. A new lasso peptide, chaxapeptin, was identified from the genome of *S. leuuwenhoekii* strain C58, it has significant inhibitory activity against human lung cancer cell line A549 [22]. Curacomycin (Figure 2a), a new antibacterial cyclic peptide, was isolated from *S. curacoi* and *S. noursei*. Likewise, a new cytotoxic peptide curacozole was reported from *S. curacoi* [23,24]. Interestingly, a rare class of ribosomally synthesized and post-translationally modified peptide, thioviridaminde was discovered from *S. olivoviridis* NA005001 [25]. The genome mining of deep sea-derived *S. atratus* SCSIO ZH16 enabled the discovery of atratumycin (Figure 2a), which is active against *Mycobacterium tuberculosis* H37Ra and H37Rv [26]. In another study, with the aid of genome mining strategies, *Streptomyces* sp. VN1 yielded diverse metabolites of non-natural furan-type anticancer compound [27]. From an extremotolerant strain, *S. huasconensis* HST28^T^, new lasso peptide, huascopeptin was identified through a genome-guided approach [28]. A novel macrolactam compound designated JBIR-156 (Figure 2a) was discovered from *Streptomyces rochei* IFO12908 through the heterologous expression of a large cryptic biosynthetic gene cluster [29]. With a slightly different approach, combining NMR-based metabolomics with genome mining, novel *C*-glycosyl-pyranonaphthoquinones (Figure 2a) were discovered from *Streptomyces* sp. MBT76 [30]. Thus, genome mining has become the primary tool for streamlining the discovery of novel compounds.

While optimization of media components increases the production of secondary metabolites to certain extent [31,32], genetic engineering is more effective [33,34]. For example, mithramycin (Figure 2b), an antitumor compound, was synthesized by manipulation of malonyl-CoA (MCoA) by carbon flux redirection in *S. argillaceus*, which increases the productivity up to 229% [35]. *S. venezuelae* was successfully engineered to upregulate the expression up to ten-fold for tylactone production [36]. In another study, enhanced flux was made possible through the shikimate pathway for the increased production of balhimycin [37]. Daptomycin is a cyclic peptide that is produced by *S. roseosporus*, and its productivity can be enhanced by 30% with the introduction of an addition copy of dptJ [38]. In addition to single biosynthetic pathways, sometimes-simple precursors were distributed simultaneously between several biosynthetic pathways. For example, bafilomycin and valinomycin are not structurally related but they share a common precursor, 2-ketoisovaleric acid, a deamination product of valine. The higher valinomycin was achieved by attenuating precursor draining away from bafilomycin biosynthesis, which increases the valinomycin production in *Streptomyces* sp. M10 [39]. The twenty-fold increase in production of actinorhodin was achieved by the recombination of RsA and RsB [40]. In another study, *S. hygroscopicus* 5008 was engineered for enhanced production of fkins A (VAL-A), a widely used antifungal agent for the treatment of sheath blight disease of rice and other plants. The increased production of VAL-A is achieved by integrating the zouA mediated DNA amplification system between the two boundaries of the val gene cluster [41]. Similarly, *S. aureofaciens* was engineered to overexpress the FADH2-dependent halogenase CtcP for chlortetracycline (CTC) production [42]. The introduction of the entire chloramphenicol gene cluster into genome-minimized *S. avermitilis* increased the production ten-fold [43]. Another example is simocyclinone biosynthesis (sim), which was achieved by amplification of the OmpR-PhoB subfamily regulator *simReg1* from *S. antibioticus*, resulting in a 2.5-fold increase [44]. Manipulation of sgCR1, sgCR2, and sgCR3 from *S. globisporus* resulted in the overproduction of enediyne antitumor antibiotic C-1027 [45]. Functional manipulations of the tetramycin regulatory gene ttmRIV enhances the production of tetramycin A and nystatin A1 in *S. ahygroscopicus* [46]. The manipulation of pathway-specific late regulator AlpW in *S. ambofaciens*, a species known to produce the congocidine and spiramycin antibiotics, leads to the synthesis of Kinamycins [47]. The overexpression of five potential target genes from *S. tsukubaensis* enhances FK506 production [48]. Recently emerging tools have made the genetic engineering process much easier. For example, in vitro TX–TL (transcription–translation) is a fast and expanding technology for the bottom-up design of complex gene expression tools, biosensors, and protein manufacturing. Based on these, *S. venezuelae* could be further expanded in its capability with the introduction of its own in vitro transcription–translation (TX–TL). The aim of this system is to provide a host for the homologous production of exotic enzymes from Actinobacteria secondary metabolism in vitro [49]. Recently, a new generation of genome engineering technologies based on a class of RNA-guided endonucleases, such as clustered regularly interspaced short palindromic repeats (CRISPR)-associated Cas9, and their rapid applications are now bringing about a further revolution in biology and medicine [50]. In particular the powerful pCRISPomyces system is amenable to the assembly of spacers and editing templates via Golden Gate Assembly and isothermal assembly [51].

## 5. Integrated Genome Mining and Metabolomics Approach

The integration of genome-mining and metabolomics through advancements made in bioinformatics and chemical analysis holds a crucial role in the field of microbial natural products discovery [52,53,54]. Nuclear magnetic resonance (NMR) and mass spectrometry (MS) coupled with ultra-high-performance liquid chromatography (UHPLC) were a matter of choice for the isolation of bioactive natural products [55,56]. In recent years, many computational tools have become available that allow automatic annotation and dereplication of metabolomics data, such as BiG-SCAPE [57], BiG-SLICE [58], RippQuest [59], molecular networking [60], and dereplication via the Global Natural Products Social (GNPS) server [61]. Comparing metabolomics results against these databases is of great value for dereplication efforts, especially for specialized metabolite discovery [62]. Based on this concept of combining genomics and metabolomics, novel non-ribosomal lipopeptide stendomycin was discovered from *S. hygroscopicus* [63]. In another study, natural products from *S. roseosporus* was mapped by integrating a molecular network and metabolomics, which led to the discovery of stenothricin [64]. Legonaridins are rare liner ribosomally synthesized and post-translationally modified peptides (RiPPs) that were discovered from *Streptomyces* sp. CT34 [65]. Furthermore, in the quest for the discovery of novel compounds we can combine the approaches of genome mining and metabolomics, which shows great promise.

## 6. Outlook

For the development of new antimicrobial drugs, genome mining becomes ad hoc, which seems to promote the discovery of novel compounds from *Streptomyces*. Notably, an upsurge in genome reports with preliminary genome screening efforts uncovers the hidden-biosynthetic potential of *Streptomyces.* Although there is a large imbalance between predicted biosynthetic potential and the rate of discovery of novel compounds, intense genome-inspired engineering work supported by well-configured genome-mining tools will drive upcoming discoveries in this field of research.

## Data Availability

Data sharing not applicable.
